# Novel Mutations in the Voltage-Gated Sodium Channel of Pyrethroid-Resistant *Varroa destructor* Populations from the Southeastern USA

**DOI:** 10.1371/journal.pone.0155332

**Published:** 2016-05-18

**Authors:** Joel González-Cabrera, Sonia Rodríguez-Vargas, T. G. Emyr Davies, Linda M. Field, Daniel Schmehl, James D. Ellis, Klemens Krieger, Martin S. Williamson

**Affiliations:** 1 Department of Biological Chemistry and Crop Protection, Rothamsted Research, Harpenden, Hertfordshire, United Kingdom; 2 ERI BIOTECMED, Department of Genetics, Universitat de València, Valencia, Spain; 3 Department of Entomology and Nematology, University of Florida, Gainesville, Florida, United States of America; 4 Bayer Animal Health GmbH, Leverkusen, Germany; University of Crete, GREECE

## Abstract

The parasitic mite *Varroa destructor* has a significant worldwide impact on bee colony health. In the absence of control measures, parasitized colonies invariably collapse within 3 years. The synthetic pyrethroids *tau*-fluvalinate and flumethrin have proven very effective at managing this mite within apiaries, but intensive control programs based mainly on one active ingredient have led to many reports of pyrethroid resistance. In Europe, a modification of leucine to valine at position 925 (L925V) of the *V*. *destructor* voltage-gated sodium channel was correlated with resistance, the mutation being found at high frequency exclusively in hives with a recent history of pyrethroid treatment. Here, we identify two novel mutations, L925M and L925I, in *tau*-fluvalinate resistant *V*. *destructor* collected at seven sites across Florida and Georgia in the Southeastern region of the USA. Using a multiplexed TaqMan® allelic discrimination assay, these mutations were found to be present in 98% of the mites surviving *tau*-fluvalinate treatment. The mutations were also found in 45% of the non-treated mites, suggesting a high potential for resistance evolution if selection pressure is applied. The results from a more extensive monitoring programme, using the Taqman® assay described here, would clearly help beekeepers with their decision making as to when to include or exclude pyrethroid control products and thereby facilitate more effective mite management programmes.

## Introduction

The ectoparasitic mite *Varroa destructor* Anderson and Trueman (Arachnida: Acari: Varroidae) causes severe damage to colonies of the Western honey bee (*Apis mellifera* L.) in many countries worldwide. It is considered to be one of the most important problems for modern apiculture and a key factor leading to the high annual losses of honey bee colonies in recent years [1], posing a serious risk for pollination. In the USA alone, the economic value of crop pollination contributed by *A*. *mellifera* is estimated to be more than 17 billion USD per year [2], while on a global scale the total value has been estimated to be 153 billion euros per annum, representing 9.5% of the world’s agricultural food production [3]. *Varroa destructor* causes direct damage to bees by feeding on the haemolymph of immature and adult bees, and it also serves as a vector for several bee viruses that contribute to a progressive decline of bee colonies [4–6]. In the absence of an effective control program, parasitized colonies will usually collapse in less than 3 years.

Currently, there is a wide array of chemotherapeutic, mechanical, cultural and behavioural management methods available for controlling the parasite (for review see [1]). However, many beekeepers tend to rely exclusively on a few active ingredients, with the synthetic pyrethroids *tau*-fluvalinate and flumethrin being amongst the most popular varroacides due to their relative low toxicity towards bees, ease of application and high efficacy, eliminating up to 98% of susceptible mites from bee colonies [7]. The application of *tau*-fluvalinate-based formulations such as Apistan® was successful for many years in controlling *V*. *destructor* levels within apiaries; however, since the mid-1990s therapeutic failures associated with the evolution of *V*. *destructor* resistance to pyrethroids have been widely reported [8–17]. Similar resistance evolution has been observed in a wide range of agricultural, veterinary and public health pests where pyrethroids, with their high arthropod selectivity, low mammalian toxicity and limited environment persistence, have likewise been used intensively [18]. Some of these cases have been attributed to alterations in the expression of certain detoxification enzymes [19], but the most common mechanism of resistance is the substitution of key residues within the voltage-gated sodium channel (VGSC), the major target site for pyrethroids [20, 21].

Recent studies have reported one particular amino acid substitution (L925V) as being associated with the resistance of *V*. *destructor* to *tau*-fluvalinate in samples collected from several locations in Central/ Southern England [22] and in the Czech Republic [23]. This leucine to valine substitution at amino acid position 925 of the VGSC, is located within transmembrane helix S5 of channel domain II (IIS5) that forms one side of a hydrophobic pocket, proposed to be the major binding site of pyrethroids within the channel protein [24]. This region of the channel, along with the IIS4-IIS5 linker and IIIS6 helix that also contribute to the binding pocket, contains a number of residues where mutations are known to give strong (so-called super-kdr) resistance to pyrethroids. These include at methionine 918 (M918T,L,V,I), leucine 925 (L925I, V), threonine 929 (T929I,C,V,N), leucine 932 (L932F), phenylalanine 1534 (F1534C) and phenylalanine 1538 (F1538I) (for review see [20]). Although other mutations have been reported from pyrethroid resistant *V*. *destructor* populations collected in Korea and the USA [14, 16], these polymorphisms do not map to regions of the channel that have been implicated in pyrethroid binding and their role in conferring resistance, if any, is not known.

In this paper, we report the identification of two alternative amino acid substitutions, L925M and L925I, in the VGSC of populations of *V*. *destructor* originating from seven different locations across the Southeastern USA. All of the *V*. *destructor* in these samples survived treatment with *tau*-fluvalinate applied at recommended field rates, with one or both of the mutations found in 98% of the resistant mites.

## Material and Methods

### Ethics statement

The samples (adult mites) used in this study were voluntarily provided by individual beekeepers knowing that the results would be used for a scientific publication.

### Varroa destructor

Honey bees were collected from managed honey bee colonies maintained by part time (50–249 colonies) or commercial (>250 colonies) beekeepers across seven different locations in the Southeastern USA between April and June 2014. The colonies were managed in Langstroth-style hives and composed of at least one full-depth Langstroth brood chamber, with additional supers added as management dictated. All colonies were managed according to practices typical of beekeeping in the region.

Approximately 3,000–5,000 bees from each of 6 colonies per location were collected from open brood frames within the brood chamber, shaken into screened plastic chambers (bee-bus.com) and transported to the Bee Biology Research Unit at the University of Florida (Gainesville, FL). The participating beekeepers did not disclose the varroacide treatment history of the colonies; however, none of the colonies had been treated within three months prior to bee collection.

At Gainesville, each screened chamber containing bees from a single colony received 100 mL of 50% (w/v) sucrose solution daily for a period of three days. For screened chambers from each location, three chambers did not receive any varroacide (negative control) and three chambers were treated with a new ¼ strip (12.25 × 1.45 cm) of Apistan (*tau*-fluvalinate) daily for three days (the used strips were removed and discarded after 24 hours, being replaced by an unused strip). The ¼ strip size per 3,000–5000 bees per chamber was chosen as the approximate labelled rate for a whole-colony treatment based upon the assumption that 40,000 bees per colony are treated with the label rate of two strips. During treatment, mites killed or dislodged by the treatment fell through the bottom of the screened chambers and were collected/counted. These were considered susceptible to the *tau*-fluvalinate treatment. All *V*. *destructor* remaining on the adult bees throughout the treatment period were considered tolerant of, or resistant to, the treatment. All chambers, including bees, were placed in a freezer at -20°C at the end of the three-day treatment period.

*Varroa destructor* that had survived the Apistan treatment were recovered following a standard method with some modifications [25]. Briefly, the frozen host bees were placed into a bucket containing 3 L of warm (~50°C), soapy water and stirred for two minutes to dislodge the mites from the bees. The mixture was then poured through a double sieve and washed with a stream of water for a period of 30 seconds. The *V*. *destructor* were quantified, placed into a 1.5mL Eppendorf tube, and immediately stored at -80°C for analysis (Table 1). *V*. *destructor* from at least one untreated control sample were analysed from each location. Not all of the *tau*-fluvalinate-treated samples had surviving mites and therefore these samples did not undergo subsequent molecular analysis.

**Table 1:** Genotyping results for individual *V*. *destructor* collected in seven locations in the Southeastern USA using the TaqMan® allelic discrimination assay.

Location^1^	Sample Name^2^	Collected mites^3^	HOM L925^4^	HOM I925	HOM M925	HET L925/I925	HET L925/M925	HET M925/I925	Total Analysed^5^
Dunnellon, FL	Treated_1	16	0	16	0	0	0	0	16
	Treated_3	8	1	7	0	0	0	0	8
	Control_1	30	15	3	4	0	2	0	24
	Control_2	30	6	0	2	0	0	0	8
Micanopy, FL	Treated_2	16	0	12	1	0	0	2	15
	Control_3	30	19	1	3	2	1	0	26
Fort Myers, FL	Treated_1	15	0	6	2	0	0	0	8
	Treated_2	22	1	11	5	0	0	1	18
	Control_2	30	11	1	9	0	4	0	25
Trenton, FL	Treated_1	5	0	3	0	0	0	2	5
	Treated_2	3	0	2	0	0	0	0	2
	Treated_3	2	0	1	1	0	0	0	2
	Control_2	19	12	0	4	1	0	0	17
Kissimmee, FL	Treated_1	14	0	11	0	0	0	0	11
	Control_2	30	11	8	3	1	2	0	25
Chipley, FL	Treated_1	7	0	4	0	0	0	0	4
	Treated_3	3	0	1	0	0	0	1	2
	Control_1	25	4	6	2	3	1	0	16
Sparta, GA	Control_2	25	13	5	6	1	0	0	25

1. Location where sample was collected.

2. Treated: Mites surviving three-day treatment with *tau*-fluvalinate. Control: mites from the same location but collected from chambers not-treated with *tau*-fluvalinate.

3. Total number of *Varroa* collected per sample.

4. Number of mites from each genotype detected in the sample. HOM: Homozygous HET: Heterozygous. L925: wild-type allele, others are mutants.

5. Total number of mites analysed.

### TaqMan® diagnostic assays

**Fig 1:**
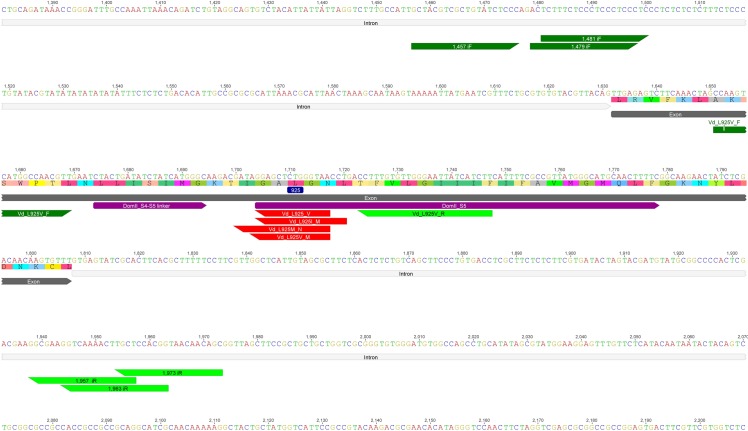
Intron-exon-intron section of the *V*. *destructor* VGSC Domain II. Section including the linker between transmembrane segments 4 and 5 (DomII_S4-S5) and the transmembrane segment 5 (IIS5) [in magenta]. Intron [light grey] and exon regions [dark grey], as well as the annealing positions for primers [green] and TaqMan® probes [red] are shown. Numbering corresponds to that in Accession KC152655. The position of the residue 925 of the channel protein is highlighted in blue.

The assays used genomic DNA extracted from individual adult *V*. *destructor* by an alkaline hydrolysis method as described previously [22] and stored at -20°C. Primers and probes for the TaqMan® assays were designed against gene sequences flanking the L925 residue of the *V*. *destructor* VGSC (Accession KC152655) (Fig 1) using Primer Express™ Software v.2.0 (Life Technologies). Primers Vd_L925V_F (5’-CCAAGTCATGGCCAACGTT-3’) forward and Vd_L925V_R (5’-AAGATGATAATTCCCAACACAAAGG-3’) reverse, were standard oligonucleotides with no modification and have been used previously in similar assays to detect L925V in European mite samples [22]. Probe Vd_L925_V (5’-TTACCCAGAGCTCC-3’) was labelled with VIC® at the 5’ end for detection of the wild-type allele (L925), probe Vd_L925V_M (5’-TTACCCAcAGCTCCT-3’) was labelled with 6-FAM™ for detection of the L925V mutation, probe Vd_L925I_M (5’-AGGTTACCtAtAGCTCC-3’) was also labelled with 6-FAM™ for detection of the L925I mutation and probe Vd_L925M_N (5’-TTACCCAtAGCTCCTATC-3’) was labelled with NED® for detection of the L925M mutation. Changes to the wild-type sequence are indicated in lower case in the probe sequences. A 3’ non-fluorescent quencher and a minor groove binder (MGB) were added to each probe. The MGB increases the Tm between matched and mismatched probes and improves the accuracy of allele discrimination [26]. TaqMan® assays were run on an ABI 7900HT Fast Real-Time PCR System (Applied Biosystems). The assay mixture contained 1.5 μl genomic DNA, 7.5 μl of 2x SensiFAST™ probe Hi-Rox Mix (Bioline Reagents Ltd), 0.9 μM each primer and 0.2μM each probe, in a total reaction volume of 15 μl. After optimization, temperature cycling conditions were set as follows: 2 min at 50°C, 10 min at 95°C followed by 40 cycles of 15 s at 95°C and 45 s at 60°C for reactions with probes Vd_L925_V and Vd_L925V_M or 62°C for reactions with probes Vd_L925_V, Vd_L925I_M and Vd_L925M_N. The increase in VIC® (538 nm excitation and 554 nm emission), 6-FAM™ (494 nm excitation and 518 emission) and NED® (546 nm excitation and 575 emission) fluorescence was monitored in real time by acquiring each cycle on the relevant filter of the ABI 7900HT.

### Analysis of sequences encoding Domain II of *V*. *destructor* VGSC

Genomic DNA from at least 2 individuals per sample was used as a template to PCR-amplify fragments covering the relevant region of domain II (Fig 1) following a two-step nested PCR strategy [27]. For the primary amplification (PCR1), the primers were 1457iF (5’-GCTACGTCGCTGTATCTCCC-3’) and 1973iR (5’-GCTGTTGTTACCGTGGAGCA-3’) and for the secondary amplification (PCR2), the primers were 1479iF (5’-ACTCTTTCTCCCTCCCTCCC-3’) and 1963iR (5’-CCGTGGAGCAAGTTTTGACC-3’). For each amplification, the reaction mixture contained 0.4 μM of each primer, 15 μl of DreamTaq Green PCR Master Mix (2×) (Thermo Scientific), 12 μl of distilled water and 1 μl of genomic DNA (or PCR 1) to a final volume of 30 μl. Cycling conditions were: 94°C for 1 min followed by 35 cycles of 94°C for 20 s, 61°C for 20 s and 72°C for 45 s, and final extension at 72°C for 5 min. The PCR fragments were precipitated with ethanol and direct sequenced (Eurofins MWG Operon, Germany) using the primers 1481iF (5’-TCTTTCTCCCTCCCTCCCTC-3’) and 1957iR (5’-AGCAAGTTTTGACCTTCGCC-3’). All primers were designed and the sequences analysed using Geneious software (Version 8.1, http://www.geneious.com/).

## Results

**Fig 2:**
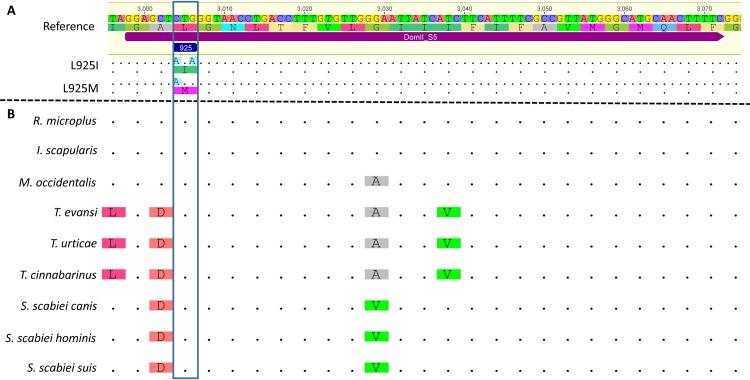
Alignment of nucleotide and/or amino acid sequences of the Voltage-Gated Sodium Channel fifth transmembrane segment (VGSC_IIS5). **A-** *V*. *destructor* reference (Accession KF771990) and mutated sequences. **B-** *Rhipicephalus microplus* (Accession Q9XZC1), *Ixodes scapularis* (Accession XP_002407119), *Metaseiulus occidentalis* (Accession XM_003741689), *Tetranychus evansi* (Accession ADK92428), *Tetranychus urticae* (Accession ADB92110), *Tetranychus cinnabarinus* (Accession ADB92494), *Sarcoptes scabiei var*. *canis* (Accession ABL11237), *Sarcoptes scabiei var*.*hominis* (Accession ABB05337), and *Sarcoptes scabiei var*.*suis* (Accession AAZ91446). Identical nucleotide and amino acid residues are shown as dots. Position 925 of the channel is boxed (*M*. *domestica* numbering EMBL accession N° X96668).

TaqMan® allelic discrimination assays designed to detect the L925V mutation that was previously described in pyrethroid-resistant *V*. *destructor* samples collected in Central/Southern England [22] gave negative results for the resistant *V*. *destructor* samples collected from across the Southeastern USA, indicating that this allele was not present in the USA samples. This negative result also suggested that, in these samples, there were different mutation(s) in the same region that might correlate with resistance. To check for these alternative mutation(s), a 485 bp gene fragment covering the domain II S4-S5 linker and IIS5 transmembrane segment of the *V*. *destructor* VGSC (Fig 1) was PCR-amplified from genomic DNA, extracted from several individuals from all samples, and sequenced. This resulted in the identification of two new polymorphisms: a single point mutation, C to A, at nucleotide 3004 of the cDNA and the same C to A substitution at 3004 together with G to A change at 3006 (as compared to the cDNA sequence of *V*. *destructor* VGSC, Genbank accession No: KF771990). These mutations result in amino acid substitutions at leucine 925; either L925M (CTG leucine to ATG methionine) or L925I (CTG leucine to ATA isoleucine) in the VGSC of *V*. *destructor*. This suggests that alternative allelic variants of L925 –M925 and I925 –are responsible for pyrethroid resistance in the USA samples, rather than the V925 allele previously reported in Europe (Fig 2A).

**Fig 3:**
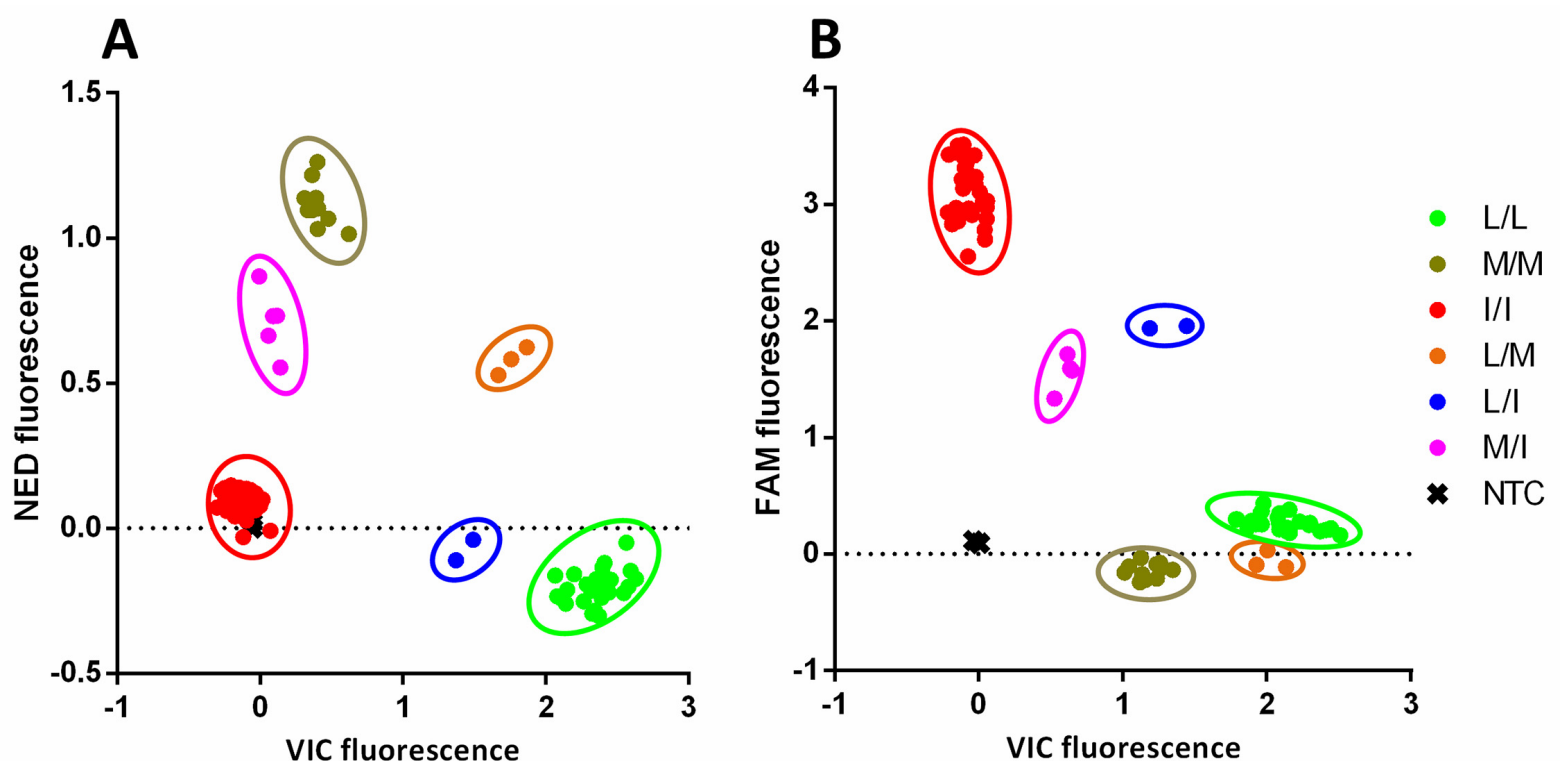
Result of an allelic discrimination TaqMan® assay for detection of mutations L925I and L925M in *Varroa destructor* VGSC. Scatter plot of fluorescence data from: **A-** NED® (M925 allele) *vs* VIC® (L925 allele) and **B-** 6-FAM™ (I925 allele) *vs* VIC® (L925 allele), showing the distribution of the six possible genotypes given the three alleles present. L/L: Homozygous for Leucine, M/M: Homozygous for Methionine, I/I: Homozygous for Isoleucine, L/M: Heterozygous for Leucine and Methionine, L/I: Heterozygous for Leucine and Isoleucine and M/I: Heterozygous for Methionine and Isoleucine. Values of X and Y axes are final corrected fluorescence data.

To determine the abundance and distribution of the 925M and 925I alleles within the USA samples, and to establish if these mutations correlate with pyrethroid resistance, new TaqMan® probes were designed to detect and discriminate these alleles in individual mites. In order to achieve this, the TaqMan® assay was multiplexed using three different custom probes, each specific for one of the three possible allelic variants L925, M925 or I925, labelled with VIC®, NED® and 6-FAM™, respectively. An increase in VIC® fluorescence would then indicate the presence of the homozygous wild-type allele (leucine), while an increase in NED® or 6-FAM™ fluorescence would indicate one of the mutant alleles, methionine or isoleucine. Intermediate increases in the fluorescence of any two of the dyes would indicate a heterozygote (L/M, L/I or M/I). After optimization, the TaqMan® assay was able to routinely discriminate among the six possible genotypes that could be generated by the three allelic variants (Fig 3).

Table 1 shows the results of the analysis of 257 individual adult mites from the 7 USA sample locations (6 from Florida and 1 from Georgia). They indicate a strong correlation (Spearman’s correlation coefficient ρ = 0.997, *P* < 0.0001) between survival of *tau*-fluvalinate (Apistan) treatment and the presence of either the M925 or I925 mutant alleles. Only two of 91 mites (2%) surviving Apistan treatment were homozygous for the wild-type allele (L925), while 74 mites (81%) were homozygous for I925 and 9 mites (10%) were homozygous for M925. A further 6 mites (7%) were heterozygous for the double mutant combination of M925/I925. For the 166 mites from untreated chambers (ie. which did not undergo selection with Apistan) 91 (55%) were homozygous for the wild-type allele, L925, and only 57 (34%) were homozygous for either of the mutated alleles. The remaining 18 (11%) were heterozygous for L925/I925 or L925/M925, and since kdr/super-kdr is a recessive mechanism, these mites would be predicted to show the susceptible phenotype.

## Discussion

A single point mutation involving the substitution of leucine by valine at position 925 of the VGSC (L925V) has been previously associated with resistance to *tau*-fluvalinate in samples of *V*. *destructor* collected in Central/ Southern England [22] and in the Czech Republic [23]. Further studies have shown that this mutation is widely distributed in *V*. *destructor* populations across Europe and is highly correlated with recent treatments with synthetic pyrethroids (our unpublished data). In contrast, we found no evidence for the L925V mutation within the samples from seven locations in Florida and Georgia despite significant resistance within these samples following treatment with *tau*-fluvalinate. Instead, we identified two alternative allelic variants at leucine 925, isoleucine and methionine (I925, M925) that correlate closely with pyrethroid resistance in these populations. Neither of these substitutions has been reported previously in *V*. *destructor*, although L925I has been identified and correlated with resistance to pyrethroids in several other pest species, including *Bemisia tabaci*, *Cimex lectularius*, *Rhipicephalus microplus*, *Trialeurodes vaporariorum* and *Hyalella azteca* [28–32]. A functional role for L925I in conferring insensitivity (and hence resistance) to pyrethroids has also been demonstrated by electrophysiological studies of *Xenopus* oocytes expressing L925I mutated channels [20, 33]. Although L925M is a novel mutation that has not been reported previously, its similarity to the other two substitutions and selection within the treated samples suggest that it too confers resistance to these compounds.

Leucine 925 is conserved across a wide range of species (Fig 2B). This amino acid is one of a small number of residues, located within the proposed binding site for pyrethroids, that are capable of mutating to give strong (so-called *super-kdr)* resistance, and also includes methionine 918, threonine 929, leucine 932 and isoleucine 936 [20, 21]. Recent modelling of the *V*. *destructor* VGSC further supports a role for these residues in the selective binding of the varroacides *tau*-fluvalinate and flumethrin, which have large, bulky acid groups that would appear to fit better into the pyrethroid binding site of mite and tick (acari) VGSCs [24, 34]. It is, however, interesting that so far, we have only found substitutions at position 925 correlating with the resistance to pyrethroids in *V*. *destructor* and as yet no variants at any of the other known resistance positions. This implies that selection pressure favours modification of this residue in *V*. *destructor*, although the reason for this is not clear and will be an interesting subject for further investigation.

In our study, most (98%) of the *V*. *destructor* surviving treatment with *tau*-fluvalinate were either homozygous for methionine (M/M), or isoleucine (I/I), or heterozygous (M/I) at postion 925 of the VGSC, but there were no L/M or L/I heterozygotes (Table 1). This is most likely a consequence of the resistance trait being recessive at the discriminating concentration of *tau*-fluvalinate present in Apistan® strips since resistance to pyrethroids is known to be a recessive trait [21]. This is important when addressing the dynamics of resistance in *V*. *destructor* with its haplo/diploid reproductive mechanism and very high levels of inbreeding [1]. The strong selection pressure imposed by a *V*. *destructor* management strategy based only on an intensive and persistent use of pyrethroids would favour a very fast spread of resistant alleles amongst populations. Since we see a very high frequency of mutant alleles in control non-treated mites (Table 1), it would be expected that a fast increase in the rate of resistant mites per bee colony would occur if they are subjected to treatment with pyrethroid-based acaricides. Our previous study also suggested a reduced fitness of *V*. *destructor* with L925V, with the frequency of resistant alleles decreasing to very low levels over several seasons when the selection pressure was removed [22]. This feature has also been reported by others [35], with bee colonies showing a 10-fold reduction in the number of resistant mites over a period of three years.

Although the use of pyrethroids for *V*. *destructor* treatment has decreased in the last 10–15 years, the results presented here and those published previously [22, 35] suggest that it is possible to design control programs that include pyrethroids in rotation with other acaricide treatments and specific cultural approaches to give effective control of the parasite. For the long-term success of such programs, it is important that the ratio of susceptible/resistant mites in a given apiary is known so that informed decisions regarding acaricide selection can be made. In this context, effective monitoring tools such as the high throughput allelic discrimination assay reported here would be particularly useful. The multiplex assay allows several alleles to be detected and monitored simultaneously and can be adapted according to the known variants within any particular region. These molecular-based tests are relatively cheap, very sensitive and significantly faster than bioassays and very importantly do not require live material. A more extensive monitoring program, widening the geographical scope of this study, would help to map *V*. *destructor* resistance in a given area and this information would be useful to regulators, companies and other stakeholders and also to beekeepers who, armed with this knowledge, would be able to judge better when they can use pyrethroid-based acaricides to control this highly damaging parasite.
